# Degenerate Human Nucleus Pulposus Cells Promote Neurite Outgrowth in Neural Cells

**DOI:** 10.1371/journal.pone.0047735

**Published:** 2012-10-16

**Authors:** Stephen M. Richardson, Devina Purmessur, Pauline Baird, Ben Probyn, Anthony J. Freemont, Judith A. Hoyland

**Affiliations:** Centre for Regenerative Medicine, Institute of Inflammation and Repair, Faculty of Medical and Human Sciences, The University of Manchester, Manchester, United Kingdom; National Cancer Institute, United States of America

## Abstract

Innervation of nociceptive nerve fibres into the normally aneural nucleus pulposus (NP) of the intervertebral disc (IVD) occurs during degeneration resulting in discogenic back pain. The neurotrophins nerve growth factor (NGF) and brain-derived neurotrophic factor (BDNF), which are associated with stimulation of axonal outgrowth and nociception by neuronal cells, are both expressed by NP cells, with BDNF levels increasing with disease severity. However the mechanism of interaction between human NP cells and neural cells has yet to be fully elucidated. Therefore the aim of this study was to determine whether non-degenerate or degenerate human NP cells inhibit or stimulate neural outgrowth and whether any outgrowth is mediated by NGF or BDNF. Human NP cells from non-degenerate and degenerate IVD were cultured in alginate beads then co-cultured for 48 hours with human SH-SY5Y neuroblastoma cells. Co-culture of non-degenerate NP cells with neural cells resulted in both an inhibition of neurite outgrowth and reduction in percentage of neurite expressing cells. Conversely co-culture with degenerate NP cells resulted in an increase in both neurite length and percentage of neurite expressing cells. Addition of anti-NGF to the co-culture with degenerate cells resulted in a decrease in percentage of neurite expressing cells, while addition of anti-BDNF resulted in a decrease in both neurite length and percentage of neurite expressing cells. Our findings show that while non-degenerate NP cells are capable of inhibiting neurite outgrowth from human neural cells, degenerate NP cells stimulate outgrowth. Neurotrophin blocking studies demonstrated that both NGF and BDNF, secreted by degenerate NP cells, may play a role in this stimulation with BDNF potentially playing the predominant role. These findings suggest that NP cells are capable of regulating nerve ingrowth and that neoinnervation occurring during IVD degeneration may be stimulated by the NP cells themselves.

## Introduction

Low back pain (LBP) is a widespread and debilitating disorder which causes a significant social and economic burden and degeneration of the intervertebral disc (IVD) has been implicated in its pathogenesis [1]. Degeneration of the human IVD is characterised by an increase in catabolic processes within the disc tissue resulting in breakdown of extracellular matrix (ECM), neoinnervation and neovascularisation [2], [3]. We and others have demonstrated the presence of nerve fibers penetrating deep into the nucleus pulposus (NP) of the painful degenerate IVD [2], [4]. Furthermore these nerve fibres have been shown to express the neural growth associated marker GAP43 and are primarily small unmyelinated neurons associated with nociception as confirmed by expression of the pain-related neuropeptide Substance P [4]. Unlike the degenerate IVD, the normal healthy IVD is largely aneural and anatomical studies by Jackson *et al* and Bogduk *et al* have demonstrated innervation of only the superficial outer layers of the annulus fibrosus (AF), with the central core of the IVD completely lacking nerves [5], [6]. However few studies have investigated the mechanisms underlying innervation into the degenerate IVD.

Johnson *et al* have investigated the effect of the IVD matrix component aggrecan on neural cell function in *in vitro* studies and have shown inhibition of neurite outgrowth in the presence of aggrecan, an effect which was abrogated after digestion of aggrecan with the matrix degrading enzyme chondriotinase [7]. The inhibitory effect of disc-derived proteoglycans on innervation has also been demonstrated *in vivo* by Melrose *et al* [8] who have shown that depletion of proteoglycans in an experimental model of disc degeneration is associated with neoinnervation into the degenerate IVD. Such studies thus suggest that proteoglycans, particularly aggrecan may play a role in suppressing nerve ingrowth.

As well as the ECM components of the IVD, soluble factors released by disc cells themselves may also be of importance. Human degenerate disc cells have been shown to produce a soluble mediator with the ability to induce neurite outgrowth from chick dorsal root ganglion [9]. The authors suggest that such a factor may belong to the neurotrophin family of growth factors which is supported by the findings that we, and others, have shown in that human degenerate IVD native cells do indeed express and synthesize the neurotrophic factors nerve growth factor (NGF) and brain-derived neurotrophic factor (BDNF) [2], [10], [11], [12]. We have also demonstrated that IL-1β, which is upregulated in degeneration [13], [14], stimulates the expression of both NGF and BDNF by NP cells, while TNF-α stimulated substance P expression [12]. Studies using a porcine subcutaneous model of disc degeneration have also highlighted a potential role for TNF-α in neoinnervation of the painful IVD [15]. These findings suggest that cytokines secreted by NP cells may act both to drive matrix degradation and to stimulate the innervation evident in degenerate discs. Indeed NGF secreted by NP cells has been shown to stimulate axonal growth and increase substance P expression by neurons from rat dorsal root ganglia [16], while intradiscal administration of anti-BDNF antibody in a rat degeneration model significantly reduced calcitonin-gene related peptide (CGRP) in dorsal root ganglia [17].

Neurotrophins are growth and survival factors, associated mainly with neuronal development, function and nociception [18]. They have the ability to induce nerve growth and axonal regeneration of peripheral nerves [19], [20]. Support for a role in innervation of the degenerate IVD comes from our previous work which has demonstrated the expression of the high affinity receptor for NGF, TrkA on ingrowing nerves [2]. Expression of the high affinity receptor for BDNF, TrkB and the low affinity NGF/BDNF receptor have also both been demonstrated to be present on cells within the IVD, including in-growing nerves [2], [11], [12].

Neurite outgrowth has been utilised by a number of researchers to investigate factors which may affect neuronal cell function. Indeed such a parameter may be useful for examining the effect of cell interactions on neuronal cell biology. The SH-SY5Y neuroblastoma cell line has been widely used as a cell culture model for investigating factors that may influence neuronal function in terms of neurite outgrowth. SH-SY5Y cells have been shown to differentiate and extend neuronal processes in response to a series of growth factors [21], [22], [23], [24]. Studies by Hynds *et al* and Snow *et al* have utilised PDGF stimulated neurite outgrowth in SH-SY5Y to investigate the effects of chondroitin sulphated proteoglycans on growth cone morphology and motility [25], [26]. Similarly, to assess the role of the chondroitin sulphated proteoglycan, aggrecan (derived from IVD tissue), on neurite outgrowth, PDGF induced neurite outgrowth from SH-SY5Y cells was used as a model [7].

The aim of the current study was to investigate human NP cell/neural interactions to identify potential mechanisms involving the release of soluble factors which may mediate nerve ingrowth into the degenerate IVD. An *in vitro* model system was established in which NP cells derived from non-degenerate and degenerate IVD tissue were co-cultured with human SH-SY5Y neural cells without cell contact. The effects of this co-culture on neurite outgrowth from SH-SY5Y cells were assessed, together with the effect of anti-neurotrophin antibodies in this system.

## Materials and Methods

### Ethics statement

Intervertebral disc tissue for use in this study was obtained with approval from both the North West Research Ethics Committee (08/H1010/36) and the University of Manchester Research Ethics Committee. Fully informed written consent from patients undergoing discectomy was also obtained.

### SH-SY5Y cell culture

SH-SY5Y cells were obtained from the European Collection of Cell Cultures and sub-cultured in eagles minimum essential medium (EMEM) and Ham's F-12 (F-12) (1∶1) supplemented with 1% v/v non-essential amino acids, 15% v/v fetal calf serum (FCS) (Life Technologies), 100 U/ml penicillin, 100 μg/ml streptomycin, 250 ng amphotericin and 2 mM glutamine (SH-SY5Y cell media). The cells were incubated at 37°C, 5% CO_2_ and media exchanged every two days.

### NP cell culture

Primary NP cells were isolated from non-degenerate and histologically degenerate [27] IVD samples obtained at post-mortem and surgical discectomy for IVD-associated chronic low back pain respectively (details of samples used are provided for each independent experiment in the relevant sections below). According to published criteria [27], all non-degenerate samples were from discs graded 0–3, while all degenerate samples were from discs graded 7–12. For cell extraction, NP tissue samples were minced and digested with 2 U/ml protease in Dulbecco's Modified Eagles Media (DMEM) and F-12 media for 30 minutes at 37°C. To isolate NP cells, IVD tissue was treated with 0.4 mg/ml collagenase type 1 (Life Technologies) for 4 hours in a shaking water bath at 37°C. The resulting cell suspension was passed through a 40 μm nylon cell strainer (BD Falcon) and the cell pellet washed twice with DMEM/F-12. Cells were then seeded in vented culture flasks and fed with DMEM/F12 supplemented with 10% v/v FCS, 100 U/ml penicillin, 100 μg/ml steptomycin, 250 ng amphotericin, 2 mM glutamine and 50 μg/ml ascorbate (NP cell media). Cells were incubated at 37°C, 5% CO_2_ and media exchanged every three to four days. Cells were used for subsequent experiments at passage <3.

### Alginate culture

Non-degenerate and degenerate NP cells were seeded in alginate to restore their native phenotype following monolayer expansion. Briefly, expanded cells were trypsinised, washed in 0.15 M NaCl and re-suspended in 1.2% w/v low viscosity alginate in 0.15 M NaCl to give a cell density of 62,500 cells per ml. The alginate/cell suspension was then expressed through a 21 gauge needle into 102 mM CaCl_2_ to form beads. Beads were then incubated in NP cell media for 9 days at 37°C, 5% CO_2_ and media was exchanged every three days.

### NP cell/neural cell co-culture

SH-SY5Y cells were trypsinised and seeded at a cell density of 25,000 cells per well of a 6 well plate in SH-SY5Y cell media overnight. Differentiation of SH-SY5Y cells was then induced by culture in serum-free SH-SY5Y cell media containing 20 ng/ml PDGF for 48 hrs at 37°C, 5% CO_2_ prior to co-culture. After 48 hours spent media was removed and six-well cell-culture inserts with a pore size of 0.4 μM were added to wells.

**Figure 1:**
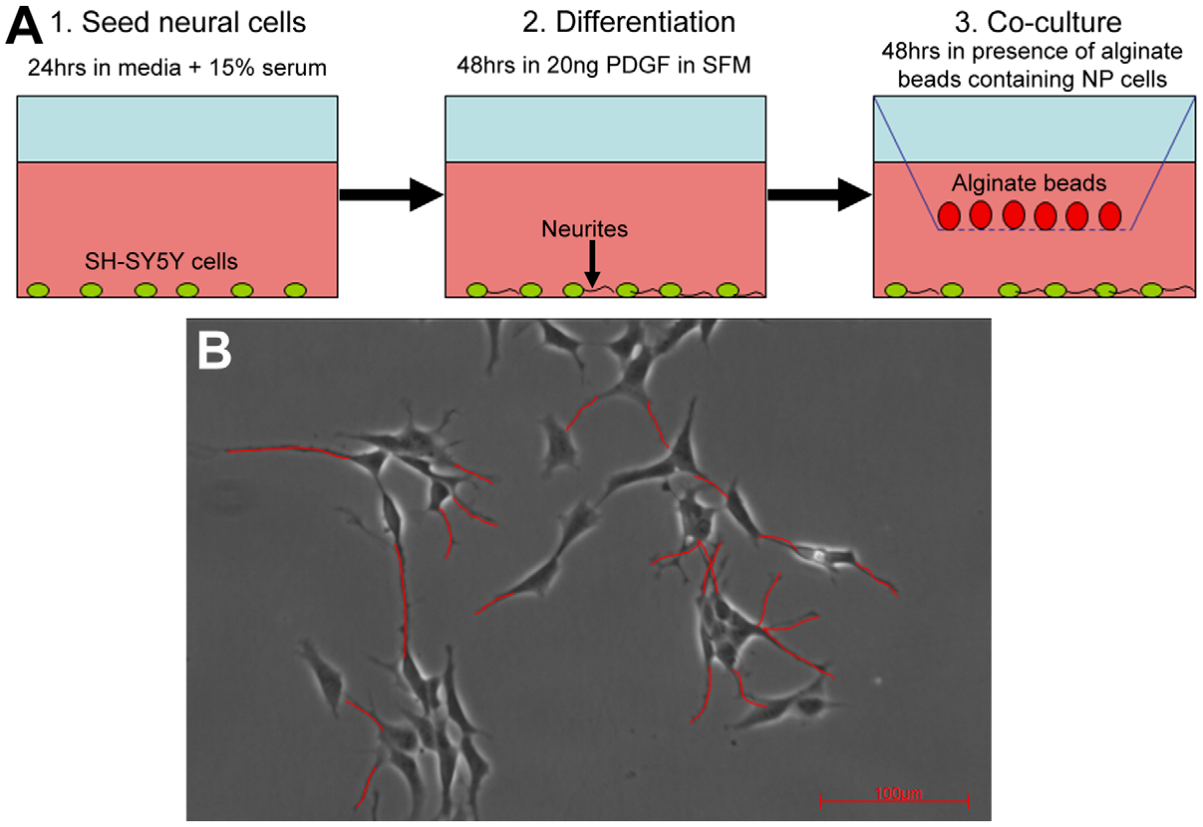
Schematic overview of experimental setup and analysis. (A) Diagram illustrating the co-culture model. (B) Measurement of neurite outgrowth from SH-SY5Y cells. Neurites were traced (red lines) and the mean neurite length measured for the total number of cells in each field of view. The mean percentage number of neurite expressing cells in each field of view was also calculated.

Alginate beads containing NP cells (n = 6 [3 non-degenerate (histological grade <3; age range 37–61 years) and 3 degenerate samples (histological grade >7; age range 27–79 years)]) were then added to each insert with serum-free SH-SY5Y cell media containing 20 ng/ml PDGF both within and surrounding the inserts (figure 1A). Co-cultures were conducted for 48 hrs at 37°C, 5% CO_2_. SH-SY5Y cells cultured in serum-free SH-SY5Y cell media containing 20 ng/ml PDGF with cell-free alginate beads served as controls (N = 6).

### Addition of anti-NGF and anti-BDNF antibodies

Co-cultures of degenerate NP cells (histological grade >7) seeded in alginate and SH-SY5Y cells were established as described above. However 1 µg/ml of anti-NGF antibody (R&D Systems) (n = 3; ages 40–51 years) or 10 µg/ml of anti-BDNF antibody (Abcam) (n = 4; ages 38–54 years) (according to manufacturer's recommendations) were added at the initiation of co-culture with appropriate controls being those cultured without antibodies.

### Analysis of neurite outgrowth

Following co-culture for 48 hours digital images of 10 fields of view (using a 10x objective lens) were taken of SH-SY5Y cells using an inverted light microscope (Leica) attached to an Infiniti X digital camera and Deltapix software. Leica Qwin software was used to assess the mean neurite length from the total number of cells expressing neurites (figure 1B). A neurite was defined (and measured) as a process extending from the cell body >20 microns in length. Only cells with both their cell bodies and processes completely within the frame were analysed. The mean neurite length per image was then averaged for the 10 fields of view to obtain average neurite length per treatment. The second parameter analysed was the percentage number of cells which expressed neurites averaged from 10 fields of view to obtain percentage number of cells expressing neurites per co-culture or control.

### Statistical analysis for neurite outgrowth data

All data was tested for normality using the Shapiro-Wilk W method of analysis. As the data did not follow a normal distribution a two-sided Mann-Whitney U test was carried to determine significance between co-cultures and controls. Values where P<0.05 were considered to be significant.

## Results

### The effect of alginate beads on neurite outgrowth

**Figure 2:**
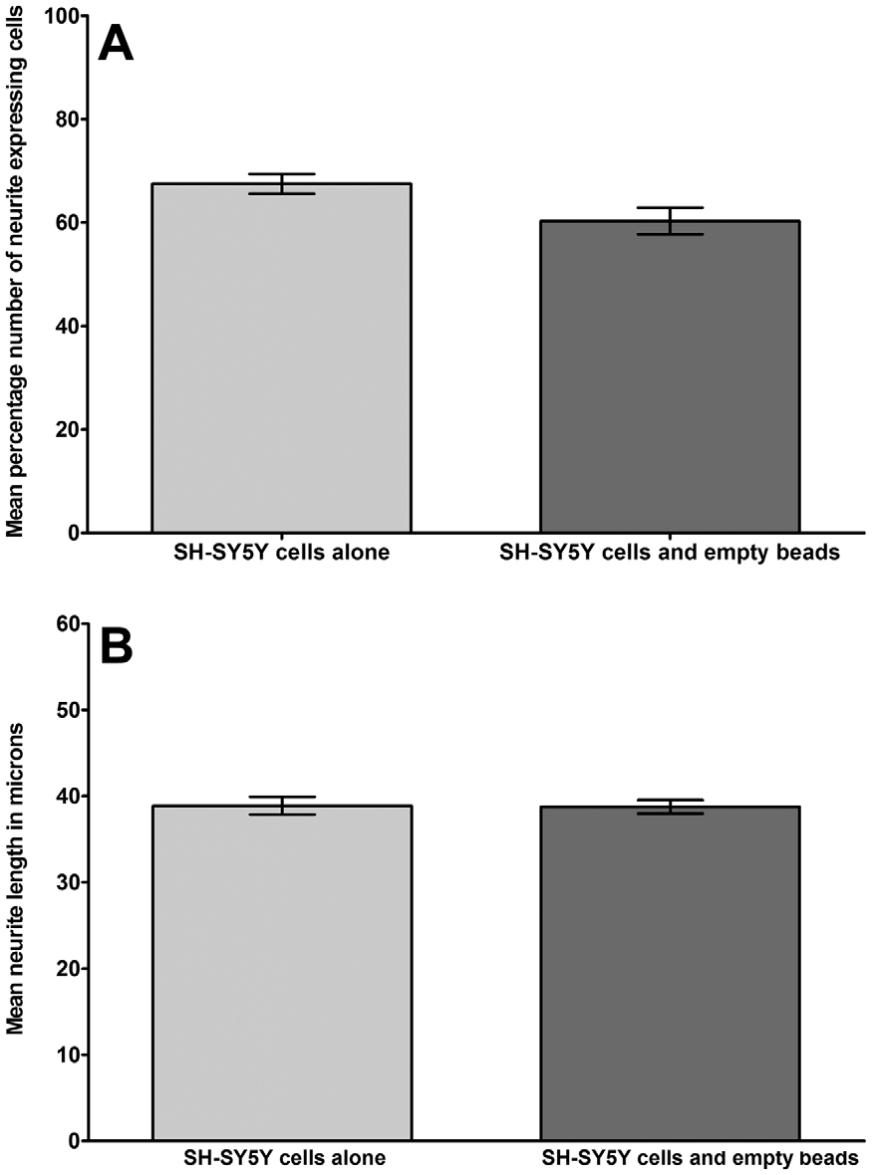
Analysis of neural cell behaviour in control cultures. Histograms to illustrate (A) the percentage number of neurite expressing cells and (B) mean neurite length from differentiated SH-SY5Y cells cultured alone, or co-cultured with empty (cell-free) alginate beads (n = 6).

Following 48 hours of co-culture mean neurite length and percentage number of neurite expressing cells was compared between SH-SY5Y cells co-cultured with empty alginate beads and SH-SY5Y cells cultured alone. Analysis revealed a slight decrease in percentage number of neurite expressing cells following co-culture (60.2%) compared to SH-SY5Y cells culture alone (67.5%), although this decrease was not significant (P = 0.08) (figure 2A). No significant (P = 0.62) change in mean neurite length was seen following co-culture with empty beads (38.74 µm) compared to cells cultured alone (38.87 µm) (figure 2B).

### Differential effects of normal and degenerate NP cells on neurite outgrowth

#### Co-culture with cells derived from non-degenerate discs

**Figure 3:**
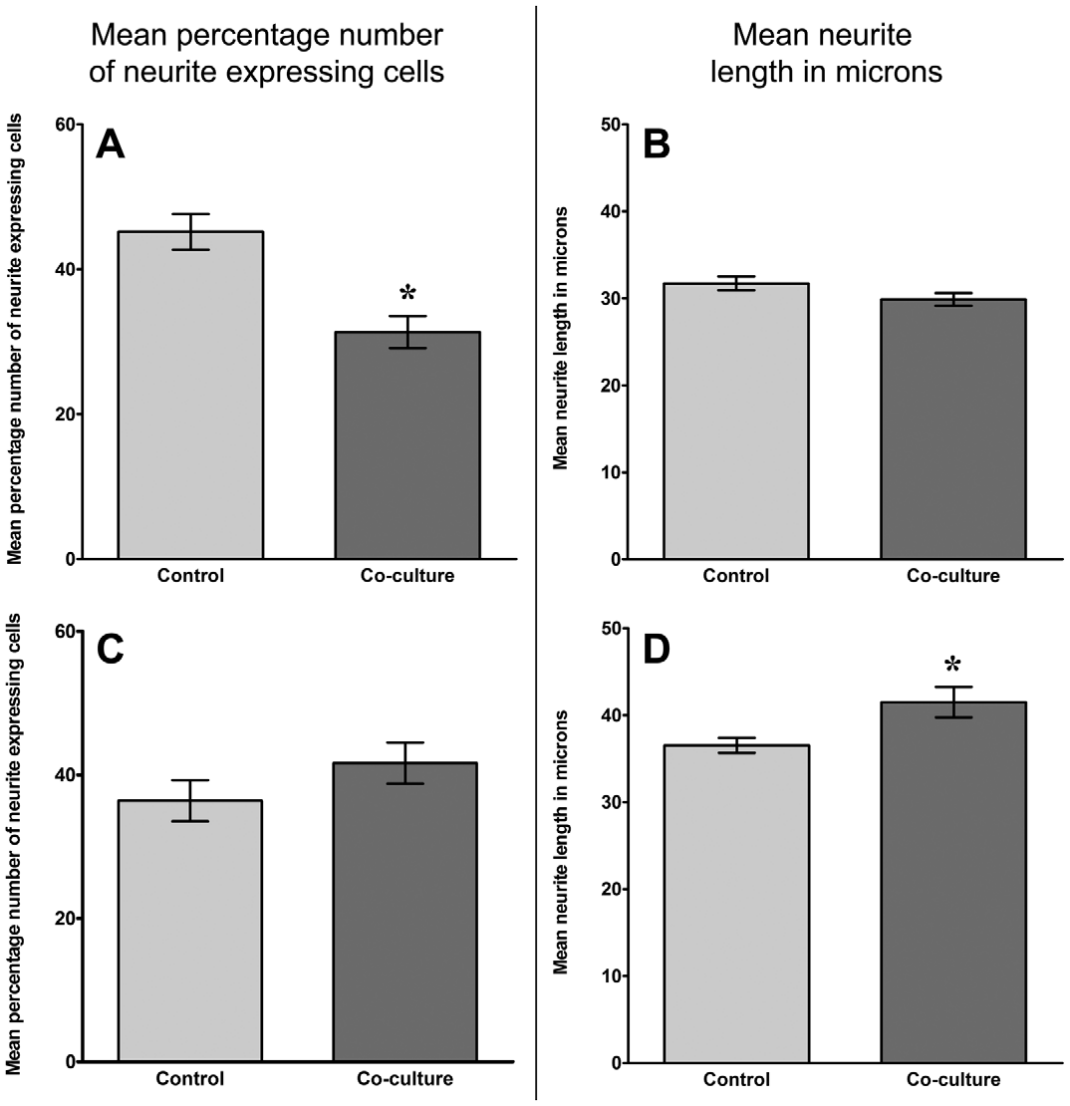
Analysis of neural cell behaviour following co-culture with normal and degenerate human NP cells. Histograms illustrating the percentage number of neurite expressing cells (A and C) and mean neurite length (B and D) from differentiated SH-SY5Y cells co-cultured with non-degenerate (A and B) and degenerate (C and D) NP cells without contact for 48 hours (n = 3 non-degenerate, n = 3 degenerate; *  = P<0.05).

After 48 hours co-culture of SH-SY5Y cells with cells derived from non-degenerate discs there was a significant (P = 0.0002) decrease in the percentage number of neurite expressing SH-SY5Y cells in the co-cultured population (31.3%) compared to SH-SY5Y cells cultured alone (45.2%)(figure 3A). When examining mean neurite length a decrease was also observed with SH-SY5Y cells co-cultured with non-degenerate NP cells (29.9 µm) when compared to its corresponding control (31.7 µm), although this decrease was not significant (P = 0.25)(figure 3B).

### Co-culture with cells derived from degenerate discs

Conversely, after 48 hours in co-culture with cells derived from degenerate IVD there was an increase in both percentage number of neurite expressing SH-SY5Y cells (figure 3C) and mean neurite length (figure 3D) compared to SH-SY5Y cells cultured alone. For percentage number of neurite expressing SH-SY5Y cells there was an increase from 36.3% to 41.6%, although this increase was not significant (P = 0.27). However, the increase in mean neurite length from 36.5 µm in controls to 41.5 μm in co-cultures cells did reach significance (P = 0.008).

### Inhibition of neurite outgrowth induced by co-culture with NP cells derived from degenerate discs

Having demonstrated an increase in percentage number of neurite expressing cells and a significant increase in mean neurite length in SH-SY5Y cells co-cultured with cells derived from degenerate IVD, the role of neurotrophins secreted by NP cells was assessed. Anti-NGF or anti-BDNF antibody was added to co-cultures and parameters of percentage of neurite expressing cells and mean neurite length assessed after 48 hours. As with the previous cohort of samples, co-culture of degenerate NP cells with SH-SY5Y cells caused an increase in both percentage number of neurite expressing cells and mean neurite length which with this cohort achieved significance in the case of percentage of neurite expressing cells (P = 0.0001), but not mean neurite length (P = 0.07).

**Figure 4:**
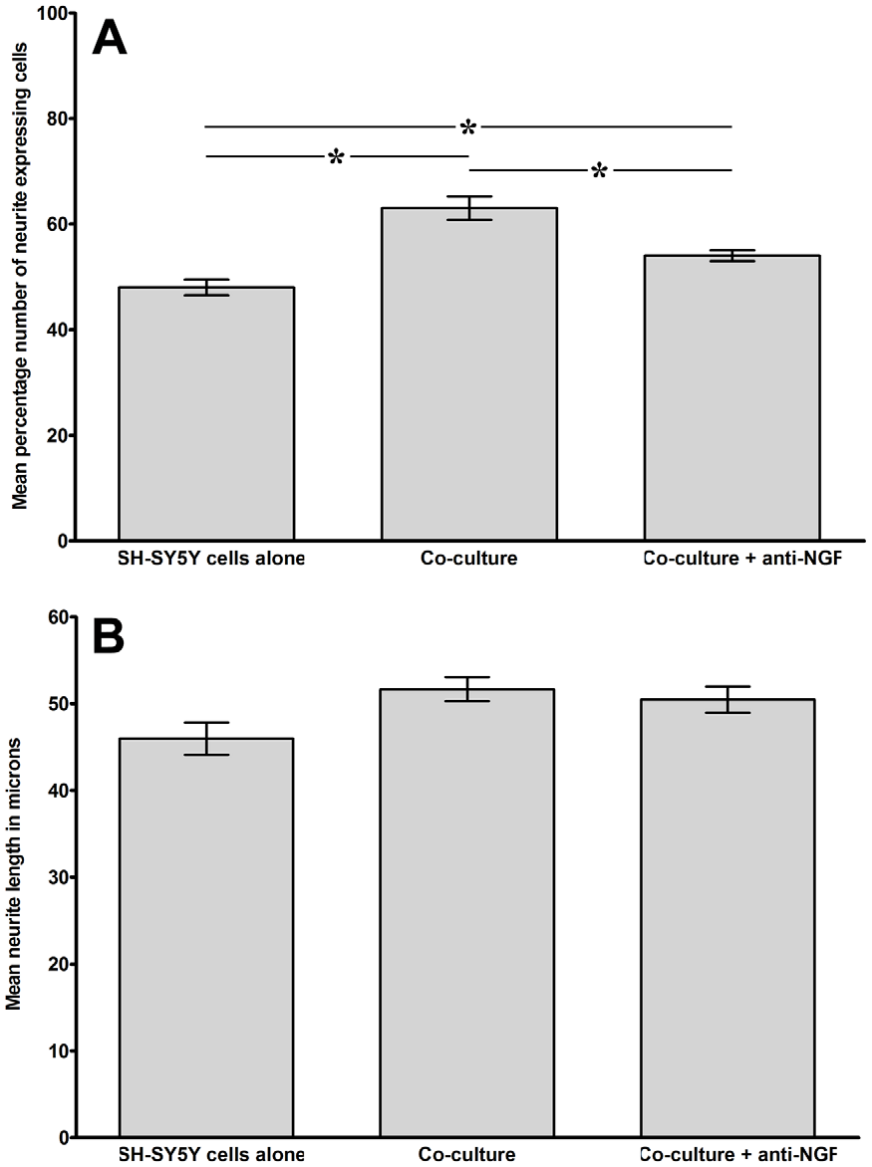
Analysis of neural cell behaviour following co-culture with degenerate human NP cells with or without addition of anti-NGF antibody. Histograms illustrating the percentage number of neurite expressing cells (A) and mean neurite length (B) from differentiated SH-SY5Y cells co-cultured with degenerate NP cells without contact for 48 hours with or without addition of anti-NGF antibody (n = 3; *  = P<0.05).

Addition of anti-NGF to the co-cultures resulted in a significant decrease (P = 0.05) in percentage number of neurite expressing cells from 63% in co-culture without blocking antibody to 54% in those co-cultures in which anti NGF was included (figure 4A). Mean neurite length was also decreased with anti-NGF relative to control co-cultures without anti-NGF (50.5 µm compared to 51.7 µm), although this decrease was not significant (P = 0.07) (Figure 4B).

**Figure 5:**
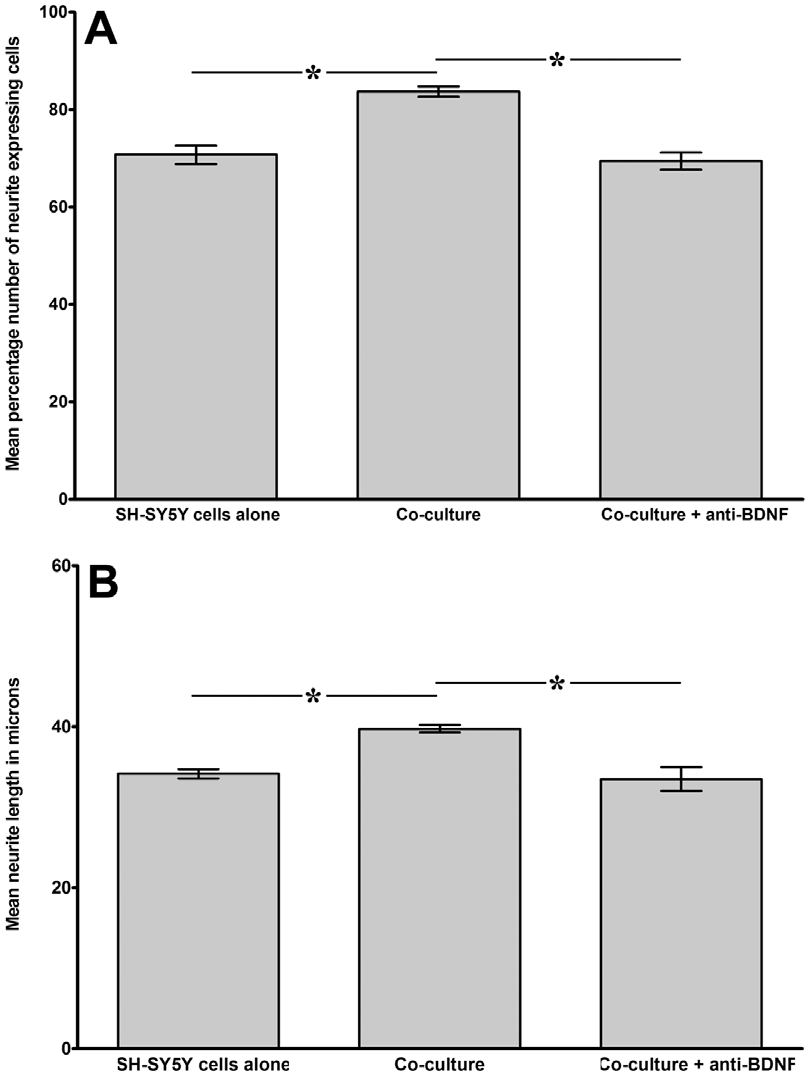
Analysis of neural cell behaviour following co-culture with degenerate human NP cells with or without addition of anti-BDNF antibody. Histograms illustrating the percentage number of neurite expressing cells (A) and mean neurite length (B) from differentiated SH-SY5Y cells co-cultured with degenerate NP cells without contact for 48 hours with or without addition of anti-BDNF antibody (n = 4; *  = P<0.05).

Co-cultures of SH-SY5Y cells and cells derived from degenerate discs were also established to test the effect of anti-BDNF in co-cultures on neurite outgrowth. Again this cohort showed similar trends to the original co-culture experiments, with significant increases in both percentage number of neurite expressing cells (71% in SH-SY5Y cells cultured alone, compared to 84% in co-cultured cells, P = 0.0054; figure 5A) and mean neurite length (34.2 µm in controls compared to 39.7 µm in co-cultured cells, P<0.0001; figure 5B). Following inclusion of anti-BDNF during co-culture the percentage number of neurite expressing cells decreased significantly (P<0.0001) to 70%, in line with control cells cultured alone. Furthermore, mean neurite length also decreased significantly (P<0.0001) to approximately those seen in control samples (33.5 µm) following addition of anti-BDNF antibody.

## Discussion

The results of this study support a role for soluble mediators released by NP cells in the mechanisms driving innervation of the degenerate IVD. NP cells derived from normal and degenerate discs had opposing effects on neurite outgrowth, with normal NP cells demonstrating inhibition of neurite length and a reduction in neurite expressing cells while degenerate NP cells showed enhancement of these parameters. Such data supports the work of Johnson *et al* who examined matrix/neural cell and disc cell/neural cell interactions and showed that normal inhibition of neurite outgrowth by aggrecan, isolated from normal discs, could be prevented by cells derived from degenerate disc suggesting that such cells release neurotrophins [7], [9]. Hence it is reasonable to suggest that matrix components and NP cells from the normal IVD exert similar effects on neuronal function and are indeed inhibitory. However, given that an indirect co-culture model was used in this study, our data suggests a possible role for soluble mediators in both inhibitory (normal NP cells) and stimulatory (degenerate NP cells) mechanisms. Interestingly we have previously demonstrated that members of the semaphorin family, which are involved in axonal guidance, are present at high levels in the normal human IVD where they are thought to play a role in inhibition of nerve ingrowth, but are significantly downregulated in the painful degenerate IVD, where nerves are present. It is therefore possible that cells derived from the non-degenerate discs used in this study produce high levels of inhibitory molecules, such as semaphorins [28], that could account for the effects reported here, although this would require further investigation. Conversely, in NP cell/neural cell co-cultures, NP cells from degenerate discs enhanced the parameters measured, suggesting they may stimulate nerve growth, which also supports the findings of Yamauchi, who demonstrated that extracted medium from NP of degenerate human IVD stimulate axonal growth in rat dorsal root ganglia [16].

While there are a wide range of soluble factors present within the IVD, including anabolic growth factors and pro-inflammatory cytokines, the most likely molecules involved in regulating nerve ingrowth during degeneration are members of the neurotrophin family. Specifically, studies have demonstrated the presence of both NGF and BDNF, as well as their receptors, within normal and degenerate IVD and NP cells have been shown to synthesise both these neurotrophins [10], [12]. Neurotrophins have been shown to stimulate axonal outgrowth from neuronal cells and thus may play a role in NP cell/neural cell interactions and nerve ingrowth into the degenerate IVD [16].

In order to assess the role of candidate neurotrophins in regulating neural cell behaviours, co-cultures were undertaken utilising blocking antibodies. In all experiments, including blocking experiments, where degenerate NP cells were used co-cultures with neural cells resulted in an increase in both the percentage of neurite expressing cells and mean neurite length. While this did not consistently reach significance the trends observed across all experiments performed were similar, strongly suggesting the release of soluble factors. The inconsistencies observed are most likely due to either the N value or to the inherent variation which occurs between human samples.

Through the addition of anti-neurotrophin antibodies to the co-culture model system, the current study has demonstrated that both NP cell-secreted NGF and BDNF may play a role in regulating neurite outgrowth. This is supported by data from Moon et al, who showed that co-culture of human AF cells with SH-SY5Y cells resulted in an increased expression of NGF, which may contribute to nerve ingrowth [29]. While in our study treatment with anti-NGF only resulted in a significant decrease in neurite expressing cells, addition of anti-BDNF antibody caused a significant decrease in both percentage number of neurite expressing cells and mean neurite length. This suggests that in our indirect co-culture system neurite growth was more dependent on BDNF. Conversely Yamauchi *et al* suggest that axonal outgrowth is dependent on NGF rather than BDNF [16], although the authors did not investigate the expression of BDNF in their system, therefore blocking with anti-BDNF may have yielded results similar to those identified here. Furthermore, the difference in findings may be due to differences in both the type of neural cells used in each study (human SH-SY5Y versus rat dorsal root ganglia) and the type of culture system employed (direct co-culture versus conditioned media). We have also previously demonstrated a significant increase in BDNF, but not NGF, expression by degenerate NP cells in line with increasing disease severity [12], again suggesting that BDNF may be the predominant factor driving neural ingrowth in degenerate discs.

Taken together these data may suggest a dysregulation in the expression of soluble mediators of neurite outgrowth, whereby in normal discs the cells act to repress neurite ingrowth through production of inhibitors such as semaphorins, but in degenerate discs the cells act to enhance neurite outgrowth through secretion of a range mediators, most notably neurotrophins such as BDNF and NGF and possibly decreased expression of semaphorin as previously reported [28]. Importantly, once the pathways involved in inhibition and stimulation of nerve ingrowth into the degenerate IVD have been fully elucidated, such inhibitory factors may be utilised in therapies which could limit nerve ingrowth and pain at the early stages of degeneration. Similarly targeting nerve growth promoting factors associated with innervation may also assist in limiting pain and nerve ingrowth into the symptomatic degenerate IVD.
